# Postural Control as a Risk Factor for Noncontact Anterior Cruciate Ligament Injury in Youth Female Basketball and Floorball Athletes

**DOI:** 10.1111/sms.70081

**Published:** 2025-05-29

**Authors:** Kattilakoski Olli, Leppänen Mari, Kannus Pekka, Steffen Kathrin, Vasankari Tommi, Kulmala Tanja, Parkkari Jari, Pasanen Kati

**Affiliations:** ^1^ Tampere Research Center of Sports Medicine UKK Institute Tampere Finland; ^2^ Faculty of Medicine and Health Technology Tampere University Tampere Finland; ^3^ Tampere University Hospital, Wellbeing Services County of Pirkanmaa Tampere Finland; ^4^ Oslo Sports Trauma Research Center Norwegian School of Sport Sciences Oslo Norway; ^5^ UKK Institute Tampere Finland; ^6^ Integrative Sport Performance Laboratory, Faculty of Kinesiology University of Calgary Calgary Alberta Canada

**Keywords:** anterior cruciate ligament injury, balance, dynamic postural control, sport injury prevention

## Abstract

The aim of this study was to investigate whether postural control was associated with an increased risk of future noncontact ACL injury in youth female basketball and floorball athletes. Data collection on 189 youth female basketball and floorball athletes was performed during a 3‐year period. The modified Star Excursion Balance Test (mSEBT), single‐leg drop‐down test, and single‐leg stance tests on a balance platform were used to measure postural control. In the mSEBT, performance in the anteromedial, medial, and posteromedial directions, as well as the corresponding composite score, were recorded. In balance platform tests, the mediolateral and anteroposterior velocity, velocity moment, and side length of a square representing 90% of postural sway were measured. Relative limb asymmetry and bilateral limb mean results for these variables were calculated and used as predictor variables in Cox regression analysis. Noncontact ACL injuries and individual exposure hours were prospectively recorded throughout the follow‐up. Twelve noncontact ACL injuries occurred. Greater limb asymmetry in the posteromedial direction [HR 1.18 (95% CI 1.05–1.32)] and composite score [HR 1.17 (95% CI 1.01–1.36)] on the mSEBT were associated with an increased risk of noncontact ACL injury. No statistically significant associations were found in the other directions for the mSEBT or any of the balance‐platform‐generated variables. Dynamic postural control, measured by limb asymmetry in the mSEBT, was associated with future ACL injury. Prevention programs for noncontact ACL injury could benefit from exercises directed toward correcting limb asymmetries in dynamic postural control in youth female basketball and floorball athletes.

## 1 Introduction

Noncontact anterior cruciate ligament (ACL) injuries and their prevention have been of great interest due to the associated prolonged absence from sports and burden on subsequent morbidity [1, 2, 3]. Moreover, it has been reported that as a consequence of ACL rupture and subsequent ACL reconstruction, only 53% of injured athletes were able to return to their previous preinjury level and that 41% of those who returned suffered an ACL reinjury to either the contralateral or previously injured leg [4].

Prior studies have shown that the majority of ACL injuries (up to 78%) and knee injuries in general are noncontact in nature [5, 6]. Noncontact ACL injuries typically occur during cutting, landing, or pivoting movements, where body weight is primarily on a single‐leg [7]. Basketball and floorball athletes frequently perform these sudden single‐leg actions. In basketball, single‐leg landings are common, while in floorball, quick single‐leg maneuvers occur during passing and shooting. Both sports also involve sudden side‐cuts and pivot turns.

The risk of ACL injury is multifactorial, meaning that it is influenced by a combination of intrinsic (e.g., anatomical, genetic, hormonal) and extrinsic (e.g., environmental, biomechanical) factors, rather than a single cause. Previous studies have indicated that nonmodifiable factors, such as female sex, previous ACL injury, and certain anatomical characteristics like bony morphology and joint laxity, may contribute to the risk of ACL injury. Additionally, modifiable risk factors, including higher BMI and reduced core and hip muscle strength, have also been suggested to play a role in increased ACL injury risk [7, 8].

Systematic video analyses on ACL injuries have shown that ACL injuries typically occur in situations in which the athlete is “off balance” at the time of the injury [9, 10]. As such, postural control has been proposed as a potential modifiable risk factor for noncontact ACL injuries [11]. However, the results of prior research have varied, and there are few studies specifically focusing on ACL injury risk [12, 13]. This highlights the need for further research on postural control as a risk factor for noncontact ACL injury.

The aim of this prospective cohort study was to investigate whether postural control is associated with the risk of sustaining a noncontact ACL injury among youth female basketball and floorball athletes. Study hypotheses formed were that scoring lower in the mSEBT or scoring higher in balance platform‐derived variables, which are thought to denote worse postural control, would increase the risk of sustaining a noncontact ACL injury [14, 15]. It was also hypothesized that increased between‐limb asymmetry in any of the postural control tests used would increase the risk for noncontact ACL injury [16, 17].

## 2 Materials and Methods

### 2.1 Study Design and Participants

The population used in this study comprised a total of six youth basketball and floorball clubs that played in the two highest youth league levels in the Tampere City district, in Finland. Only female athletes were included in the current analysis. The participating athletes signed a written informed consent form, with a parent's or guardian's consent being acquired from athletes under the age of 18, before taking part in the study. The study is part of the Predictors of Lower Extremity Injuries in Team Sports (PROFITS) study, for which the in‐depth protocol has been described elsewhere [18]. Sample size calculations were made based on ACL injury incidences for this larger cohort study, which included both female and male team sport athletes, rather than this current analysis [18]. The goal was to have a population with 20–50 injuries to be able to detect moderate to strong associations between risk factors and injury risk according to the article by Bahr and Holme [19]. This study was conducted in accordance with the Declaration of Helsinki and approved by the Ethics Committee of the Pirkanmaa Hospital District, Tampere, Finland (ETL‐code R10169).

**FIGURE 1:**
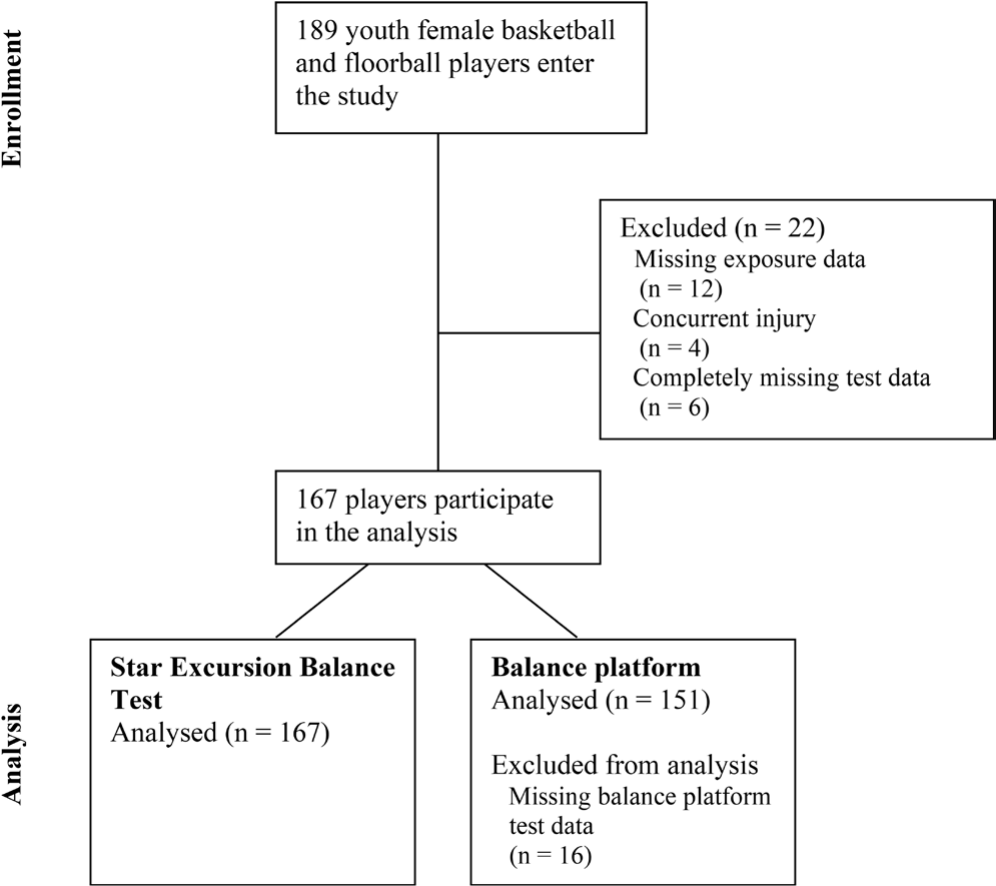
Flow chart of study participants.

One hundred and eighty‐nine athletes entered the study during the three study years (87, 43, and 59 athletes in 2011, 2012, and 2013, respectively). The date range for enrollment ranged from May 4, 2011, to May 29, 2013. Of these, 22 athletes were excluded (six due to missing postural control test data, four due to injury at baseline, and twelve due to missing exposure data). As such, 167 athletes were analyzed using the mSEBT data. Of the analyzed athletes, 93 were from basketball and 74 from floorball. Balance platform test data was analyzed separately using a smaller subset of 151 athletes, as an additional 16 athletes were excluded due to missing balance platform test data. The flow chart of study participants is shown in Figure 1. The athletes were tested at the start of their enrollment and followed for new noncontact ACL injuries for one to 3 years.

### 2.2 Testing Procedures

The physical testing of the athletes was carried out at Tampere Research Center of Sports Medicine's testing laboratory. Teams were invited to participate at one of the testing dates annually, and the full testing protocol was carried out during a single day. Tests were performed individually and included three‐dimensional motion analyses, lower body strength tests, and flexibility testing, in addition to the postural control tests reported here [18].

Anthropometric measurements were taken, including total height and leg length, which were measured as the distance from the floor to the hip joint center. Tests for postural control included three direction mSEBT as well as single‐leg drop‐down and single‐leg stance tests done with Good Balance force platform (Good Balance, Metitur, Jyväskylä, Finland) [13, 18, 20, 21].

### 2.3 Modified Star Excursion Balance Test

**FIGURE 2:**
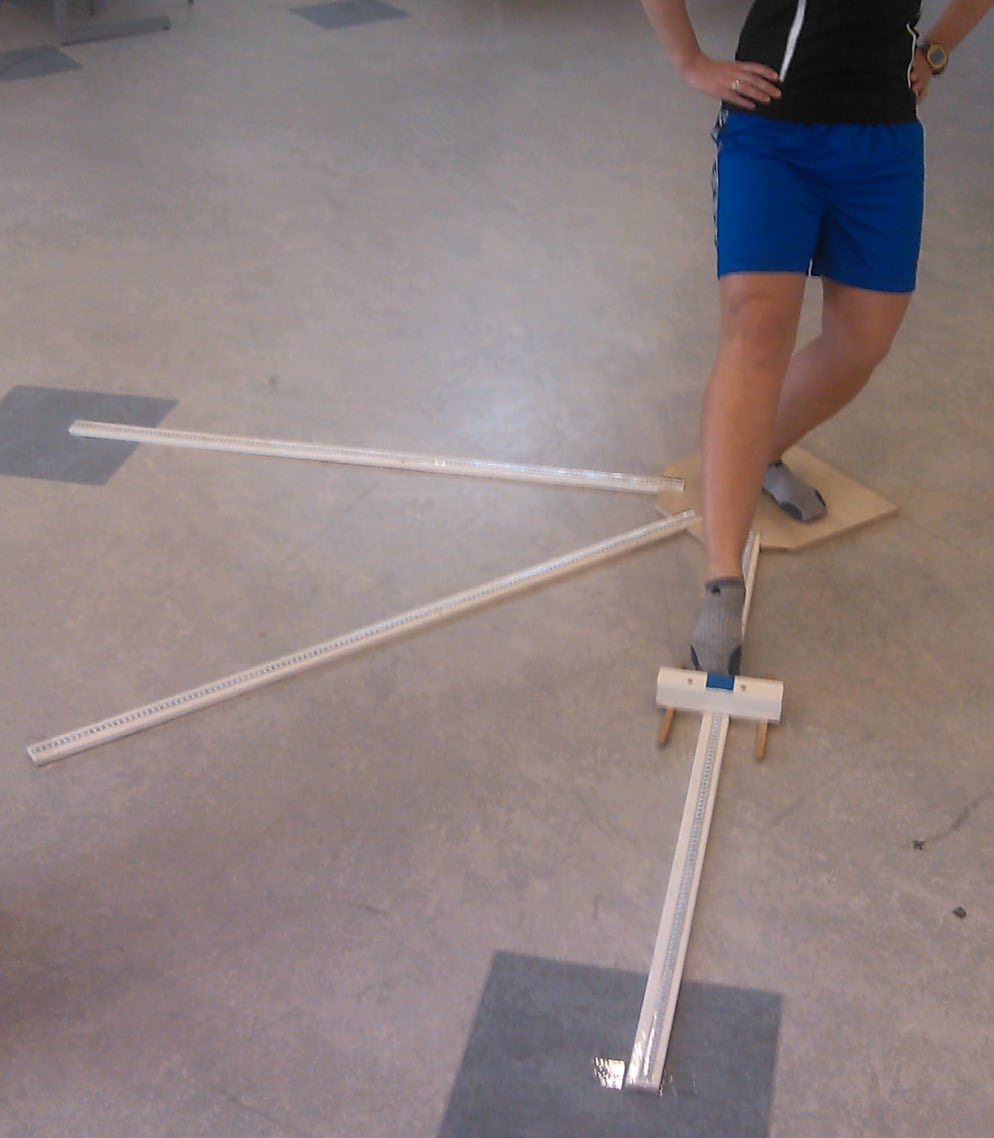
Execution of the modified Star Excursion Balance Test in the anteromedial direction.

Modified SEBT, with anteromedial, medial, and posteromedial reach directions as suggested by Hertel et al., was used to assess dynamic postural control of the athletes [13, 18, 22]. SEBT in general has been regarded as having good reliability and validity in measuring dynamic postural control [14, 23, 24]. In the mSEBT, athletes were instructed to stand barefoot on a single‐leg, with the standing foot placed in the center of the test area. From this starting position, the athlete was to reach out with the contralateral leg, sliding a wooden measurement block on a measurement tape as far as possible in the anteromedial, medial, and posteromedial directions and returning to the starting position between each repetition (Figure 2).

The athlete began with the self‐reported dominant leg based on a question regarding which foot they would use to kick a ball. The athlete had to keep the heel of their standing foot on the ground, as well as keep both hands on the iliac crests during the entire test. Athletes were not allowed to shift weight onto the outstretched limb. The athletes had one practice trial in every direction, and the following test included three successful trials in each direction. The trial was determined to be successful if the hands remained on the iliac crests and the athlete returned to the starting position without touching the ground. The distance of each trial was marked down by the test administrator in centimeters. In mSEBT, scoring higher in the test is regarded as representing better postural control [14]. Measurements were adjusted for contralateral (reaching) leg length displayed as percentages [(variable/contralateral leg height (cm)) × 100]. An additional composite score was calculated as the mean of the scores in the three test directions. The mean of three trials was calculated for each test direction and the composite score. The final variables considered in the analysis were generated using these averages. We calculated relative limb asymmetry using the bilateral ratio (higher‐scoring leg's result/lower‐scoring legs result). The bilateral mean of both legs was also calculated [(Leg_1_ + Leg_2_)/2].

### 2.4 Balance Platform Tests

This study used the single‐leg stance test and the single‐leg drop‐down test on a balance platform to assess static postural control and stabilization ability, respectively [13, 18]. Reliability of tests such as the single‐leg stance test has been examined to range from moderate to good, while the reliability of more novel tests such as the single‐leg drop‐down test is still to be determined [15].

**FIGURE 3:**
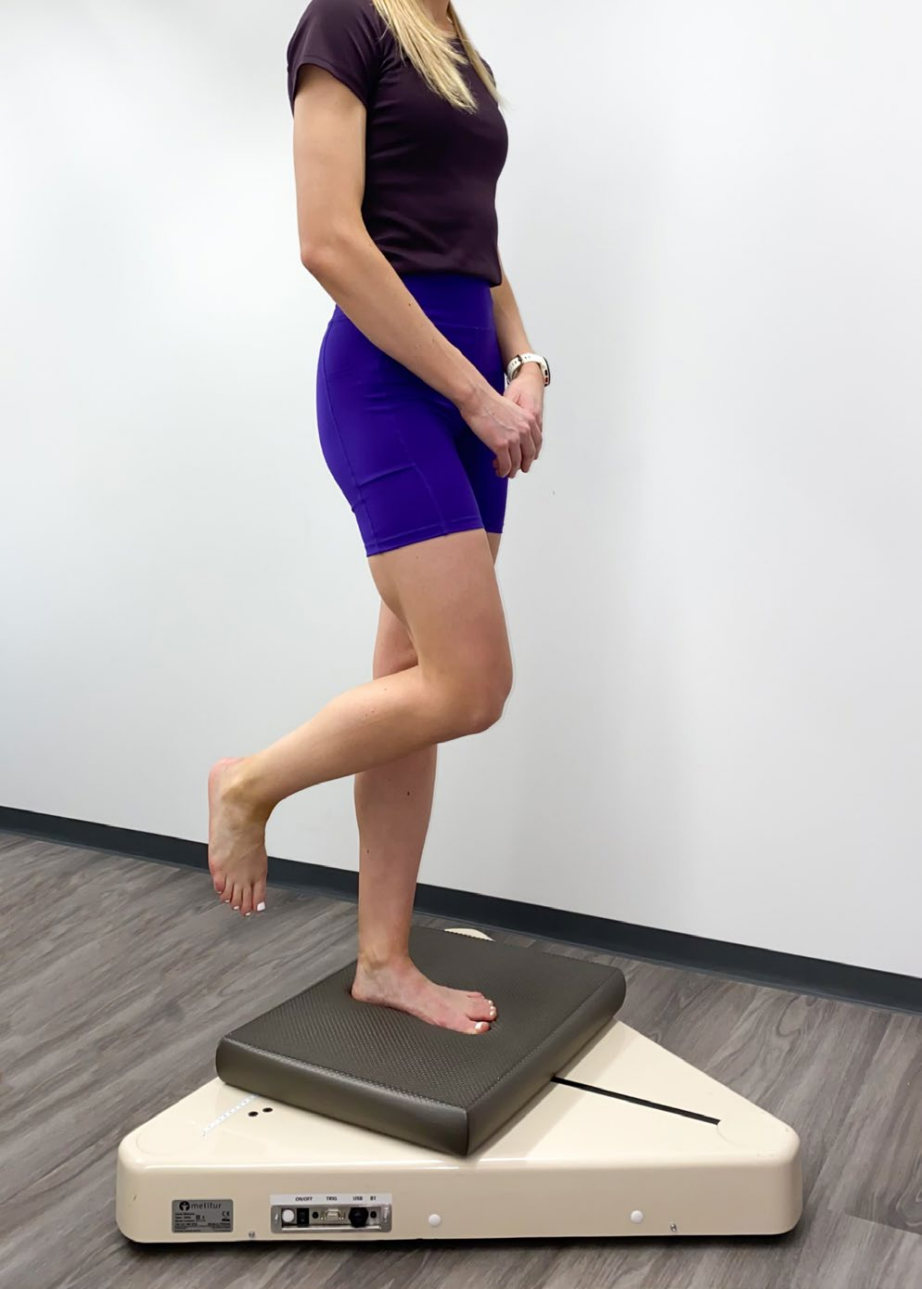
Execution of the single‐leg stance test.

In the single‐leg stance test, the athlete stood barefoot with one foot on a balance pad (Airex Balance Pad Elite, 48 cm × 40 cm × 6 cm, Alcan Airex, Sins, Switzerland) that was placed on the balance platform. The athlete held their arms in a relaxed position in front of the body. Athletes were instructed to look straight forward (Figure 3). Postural swaying was evaluated using the center of pressure (COP), which was measured using the balance platform for 20 s per trial. Athletes were allowed one practice trial, followed by as many trials as needed to reach three successful trials per leg. A trial was deemed successful if the athlete was able to complete the test without having contact with the ground, balance pad, or contralateral leg.

**FIGURE 4:**
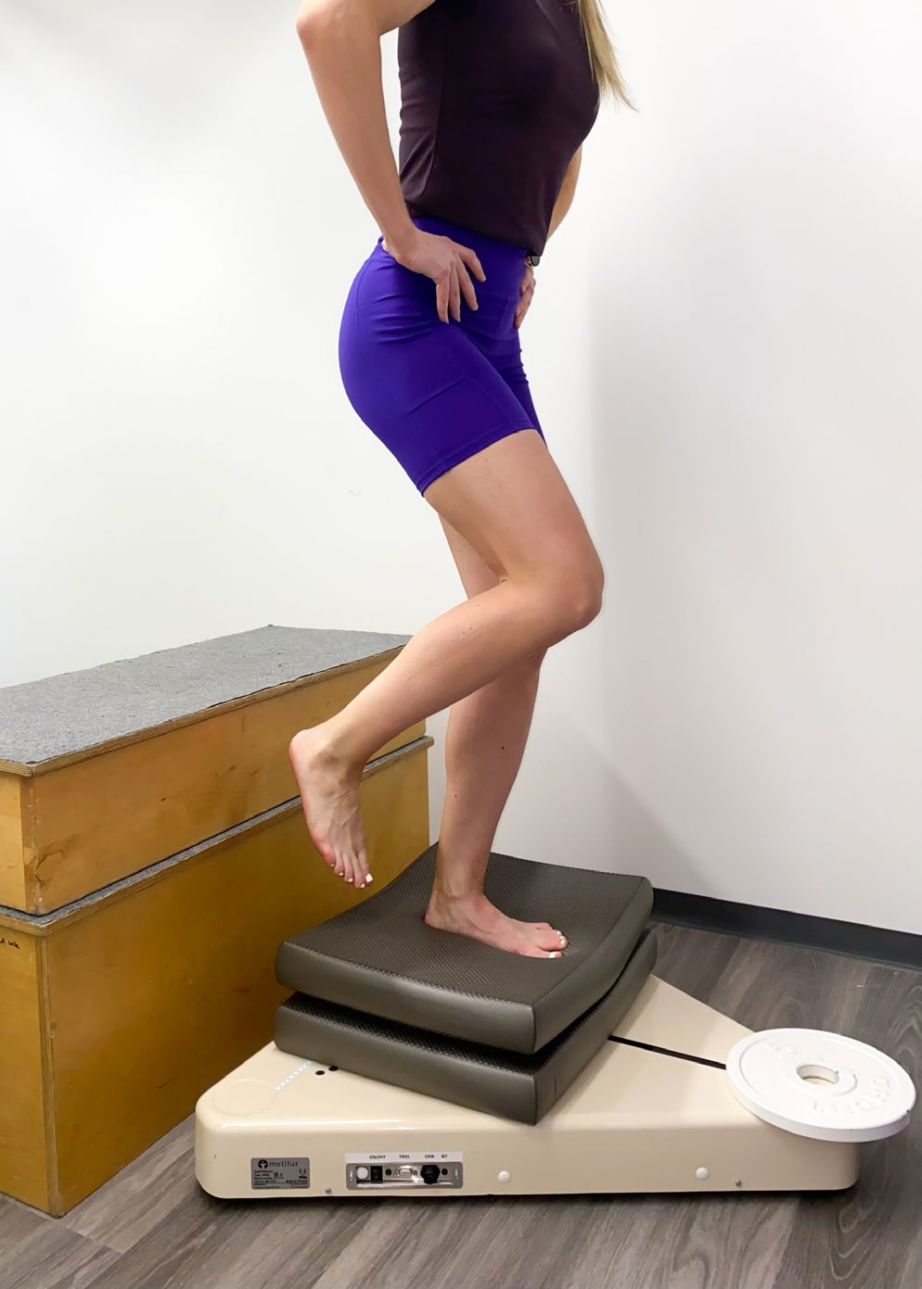
Execution of the single‐leg drop‐down test.

In the single‐leg drop‐down test, the same balance platform was used to measure postural swaying, with the athlete standing barefoot on one foot on a 60‐cm‐high bench that was placed behind the platform. Two balance pads were situated one on top of the other on the platform with the resulting height difference between the bench and the platform, and as such the height of the drop, being 30 cm. A 5‐kg weight disc was placed on the peak of the triangular platform to compensate for the impact of the drop. The athlete dropped down on the pads, landing on the same single‐leg and attempting to stabilize their position for 5 s. The athletes were instructed to hold their hands on their iliac crests during the test (Figure 4). One practice trial was followed by as many trials as required to reach five successful trials with each leg. The first three successful trials were used for the analysis. A trial was deemed successful if the athlete remained on the platform without touching the ground or the platform with the contralateral leg and their hands remained on their iliac crests.

The variables collected from the balance platform were the average mediolateral velocity of postural swaying (mm/s), the average anteroposterior velocity of postural swaying (mm/s), and the side length of a square that includes 90% of postural swaying (mm) and velocity moment (mm^2^/s). In balance platform tests, lower scores in the collected variables are considered indicative of better postural control [15]. The measurements were adjusted for athlete height using [(variable/athlete height (cm)) × 180], except for velocity moment [(variable/athlete height (cm)^2^) × 180^2^]. The mean of three successful trials was calculated and used to generate relative limb asymmetry using bilateral ratio (higher‐scoring limb's result/lower‐scoring limbs result), as well as the bilateral mean variables that were used in the analysis.

### 2.5 Injury and Exposure Registration

During the follow‐up period, the study physiotherapist contacted the coaches of the teams weekly about potential injuries. All injuries that resulted in an inability to fully participate in a game or practice session during at least the following 24 h were recorded. If an injury was reported, the injured athlete was interviewed over the phone by a study physician using a structured questionnaire. The definition of noncontact ACL injury was a magnetic resonance imaging or arthroscopy‐confirmed ACL rupture that had occurred during scheduled team practices or games with no direct contact or strike to the affected leg at the time of injury. Team coaches recorded their athletes' individual practice and game minutes on a team diary and submitted the data to the study physiotherapist on a monthly basis. Other types of injuries sustained during the follow‐up did not affect the analysis with the exception of reducing the individual athlete exposure time if the athlete could not participate in a game or practice session due to the injury.

### 2.6 Statistical Analysis

A Mann–Whitney *U* test was used to determine whether there were statistically significant differences in athlete characteristics between the injured and non‐injured athlete groups. A Cox regression analysis, which was conducted using the R programming language (R Core Team), was used to calculate proportional hazard ratios (HRs). A *p*‐value under 0.05 was used as a determinant of statistical significance, and 95% confidence intervals were calculated for the hazard ratios. An athlete's first available test data were used, and exposure time was collected until noncontact ACL injury or the end of the follow‐up period. Athletes' sport clubs were added into the model as a random factor. The need to include athlete age and earlier ACL injury in the model was tested using a *p*‐value of under 0.2 as the limit of inclusion, and this was deemed unnecessary.

A receiver operating characteristics (ROC) curve analysis, which was conducted using IBM SPSS Statistics 28 (International Business Machines Corporation), was calculated to assess the combined sensitivity and specificity of a test when a significant association between the postural control variable and the outcome was found. The results of the ROC analysis were defined as excellent (0.90–1.00), good (0.80–0.89), fair (0.70–0.79), poor (0.60–0.69), or failing (0.50–0.59).

## 3 Results

### 3.1 Participant Characteristics

A total of 58 098 h of exposure was collected. Twelve noncontact ACL injuries were sustained during the follow‐up. Mean time between testing and injury was 46 weeks. Two athletes had two noncontact ACL injuries during follow‐up, one of which was to the contralateral limb, but exposure collection ended at the primary injury as per protocol. Fifty‐seven athletes were lost to follow‐up (two due to injury, four due to moving to another city, twelve due to changing sport club and thirty‐nine due to retiring from sport). Data from drop‐outs is included for the time they participated.

The incidence of noncontact ACL injury was 0.21 per 1000 h of exposure. Noncontact ACL injury incidence was also calculated separately for floorball and basketball, which had incidences of 0.26 and 0.13 injuries per 1000 h of exposure, respectively. The incidence rate ratio between the sports was 2.05 (95% CI 0.58–9.40) which was not statistically significant (*p*‐value 0.29). Three study athletes had previous ACL injury, all of whom remained in the non‐injured group. No statistically significant differences between the injured and non‐injured athlete groups were found based on the selected descriptive characteristics (Table 1).

**TABLE 1:** Study participant characteristics.

Participants	Age (years, SD)	Height (cm, SD)	Weight (kg, SD)	BMI (kg/m^2^, SD)	Years in sport (years, SD)	Previous ACL‐injury (*n*)
Participants (*N* = 167)	15.2, 1.6	167.4, 6.2	60.4, 8.2	21.5, 2.5	6.2, 2.4	3
Non‐injured (*n* = 155)	15.2, 1.6	167.5, 6.3	60.4, 8.2	21.5, 2.5	6.2, 2.4	3
Injured (*n* = 12)	15.7, 1.7	165.7, 5.1	61.5, 7.9	22.4, 2.4	6.2, 2.5	0

*Note:* Values are presented as means except for previous ACL‐injury.

### 3.2 Regression Analyses

#### 3.2.1 Star Excursion Balance Test

Increased relative limb asymmetry was identified as a risk factor for noncontact ACL injury. This was true both in the posteromedial direction [HR 1.18 (95% CI 1.05–1.32)] and with the composite score [HR 1.17 (95% CI 1.01–1.36)] (Table 2). Asymmetry was not significantly associated with injury in the other test directions. The injured leg was the higher‐scoring limb in 5 out of 12 cases (42%) in the posteromedial direction and in 8 out of 12 cases (67%) in the composite score.

**TABLE 2:** Modified Star Excursion Balance Test's association with noncontact ACL injury risk, as analyzed by Cox regression.

Variable	Non‐injured, mean (SD)	Injured, mean (SD)	HR	95% CI	*p*
Modified Star Excursion Balance Test					
Between‐limb asymmetry (ratio)					
Anteromedial	1.03 (0.02)	1.04 (0.04)	1.07	(0.89–1.29)	0.480
Medial	1.04 (0.04)	1.06 (0.05)	1.06	(0.94–1.20)	0.350
Posteromedial	**1.04 (0.03)**	**1.07 (0.07)**	**1.18**	**(1.05–1.32)**	0.007*
Composite score	**1.03 (0.03)**	**1.05 (0.04)**	**1.17**	**(1.01–1.36)**	0.039*
Bilateral limb mean					
Anteromedial (%)	90.4 (4.8)	88.3 (4.0)	0.92	(0.82–1.03)	0.150
Medial (%)	90.2 (7.3)	87.7 (7.2)	0.94	(0.87–1.02)	0.120
Posteromedial (%)	96.6 (9.7)	95.2 (9.5)	0.96	(0.90–1.02)	0.170
Composite score (%)	92.4 (6.7)	90.4 (6.2)	0.93	(0.86–1.02)	0.120

*Note:* Modified Star Excursion Balance Test data are shown as % of leg length. “Bold” and “*” indicates statistically significant results.

Abbreviations: CI, confidence interval; HR, Hazard ratio per 1 unit of difference except for between‐limb asymmetry (per 0.01 units of difference); SD, Standard deviation.

No statistically significant associations were found for the bilateral mean results. The ROC curve analysis of between‐limb asymmetry in the posteromedial direction and composite score showed area under the curve (AUC) values of 0.611 (95% CI 0.443–0.780, *p*‐value 0.20) and 0.663 (95% CI 0.525–0.802, *p*‐value 0.02), respectively, indicating poor combined sensitivity and specificity for the test. The AUC value was only statistically significant for the composite score.

#### 3.2.2 Balance Platform Tests

No statistically significant associations with noncontact ACL injury risk were found for either the asymmetry or bilateral mean results on the single‐leg drop‐down test (Table 3). Increased relative limb asymmetry in terms of mediolateral avg. velocity [HR 1.03 (95% CI 1.00–1.07)], anteroposterior avg. velocity [HR 1.02 (95% CI 1.00–1.05)], and square side length [HR 1.01 (95% CI 1.00–1.03)] was near statistical significance, with *p*‐values of 0.07, 0.06, and 0.08, respectively. No statistically significant associations with noncontact ACL injury risk were found for either the asymmetry or bilateral mean results on the single‐leg stance balance test (Table 3).

**TABLE 3:** Balance platform tests association with noncontact ACL injury risk, as analyzed using Cox regression.

Variable	Non‐injured, mean (SD)	Injured, mean (SD)	HR	95% CI	*p*
Balance platform tests					
Single‐leg drop‐down test					
Between‐limb asymmetry (ratio)					
ML avg. velocity	1.17 (0.16)	1.22 (0.14)	1.03	(1.00–1.07)	0.072
AP avg. velocity	1.19 (0.20)	1.28 (0.20)	1.02	(1.00–1.05)	0.063
Square side length	1.21 (0.32)	1.39 (0.36)	1.01	(1.00–1.03)	0.075
Velocity moment	1.33 (0.43)	1.41 (0.45)	1.00	(0.99–1.02)	0.550
Bilateral limbs mean					
ML avg. velocity ((mm/s)/cm)	52.0 (10.2)	50.3 (6.5)	0.99	(0.93–1.05)	0.670
AP avg. velocity ((mm/s)/cm)	63.0 (12.0)	62.6 (6.7)	0.98	(0.93–1.04)	0.560
Square side length (mm/cm)	51.9 (10.2)	52.7 (7.4)	1.00	(0.93–1.06)	0.940
Velocity moment (mm^2^/cm)	500.8 (162.4)	486.5 (89.5)	0.99	(0.95–1.03)	0.700
Single‐leg stance balance test					
Between‐limb asymmetry (ratio)					
ML avg. velocity	1.17 (0.15)	1.11 (0.10)	0.99	(0.94–1.04)	0.600
AP avg. velocity	1.14 (0.13)	1.14 (0.13)	1.00	(0.95–1.05)	0.980
Square side length	1.16 (0.17)	1.13 (0.13)	1.00	(0.95–1.05)	0.940
Velocity moment	1.28 (0.29)	1.17 (0.23)	1.00	(0.97–1.03)	0.370
Bilateral limbs mean					
ML avg. velocity ((mm/s)/cm)	29.6 (6.5)	27.6 (5.4)	0.95	(0.85–1.06)	0.340
AP avg. velocity ((mm/s)/cm)	27.0 (5.4)	25.6 (5.2)	0.96	(0.86–1.08)	0.540
Square side length (mm/cm)	33.3 (5.7)	32.0 (3.9)	0.96	(0.85–1.08)	0.480
Velocity moment (mm^2^/cm)	156.1 (52.3)	138.4 (35.9)	0.93	(0.80–1.07)	0.300

*Note:* Balance platform data were normalized for athlete height.

Abbreviations: 95% CI, 95% confidence interval; AP, anteroposterior; HR, Hazard ratio per 1 unit except for bilateral limbs mean result of Velocity moment (per 10 units of difference) and for between‐limb asymmetry (per 0.01 units of difference); ML, mediolateral; SD, Standard deviation.

## 4 Discussion

This study investigated whether postural control test variables were associated with noncontact ACL injury risk in youth female basketball and floorball athletes. Increased limb asymmetry in the posteromedial direction and the composite score derived from the anteromedial, medial, and posteromedial directions of the mSEBT were identified as risk factors for ACL injury. However, the mSEBT cannot be used alone to predict ACL injury due to its low combined sensitivity and specificity. Measuring postural swaying using a balance platform did not improve injury prediction.

Steffen et al., using the same postural control tests, did not find an association between the postural control test results and noncontact ACL injury risk in elite female handball and football athletes [13]. However, Steffen and colleagues did not investigate between‐limb asymmetry, which we found to be the most important factor related to ACL injury risk. The cohorts also differed between the studies, as Steffen and colleagues studied adult elite female athletes, as compared to the youth athletes of this study. As such, there were some differences in the mean test results, with youth athletes in this study having higher mean mediolateral and anteroposterior velocities on balance platform tests and similar mSEBT results.

DuPrey and colleagues have previously identified a longer time to stabilization after a single‐leg backwards jump as a risk factor for noncontact ACL injuries in college team sports [12]. In addition, Oshima et al. identified poor static balance, measured with a gravicorder, during a two‐leg eyes open standing test as a risk factor for noncontact ACL injury [11].

While there is a scarcity of studies regarding dynamic postural control and the risk of noncontact ACL injury, there have been multiple studies done on associations between SEBT variants and lower extremity injuries. Poor dynamic postural control performance measured by composite score in the Y‐Balance test variant of the SEBT has been previously associated with elevated risk for noncontact lower extremity injury in male college football athletes by Butler et al. [25]. In a study by Plisky et al. on high school basketball athletes, a lower composite score using the anterior, posteromedial, and posterolateral SEBT reach directions was also associated with increased lower extremity injury risk for female athletes. Additionally, they found increased anterior reach asymmetry to be associated with increased lower extremity injury risk [23]. Gonell et al. found increased posteromedial reach asymmetry to be associated with lower extremity injury risk among male professional soccer players, complementing our study findings [26].

There is a lack of comparable incidence rates in the current literature for noncontact ACL injuries in youth female basketball and floorball athletes [27]. However, this study's incidence rate for noncontact ACL injury in youth female floorball athletes is similar to the one reported by Pasanen et al. for adult female floorball athletes [28].

Training programs proven effective in preventing ACL injuries have typically incorporated a postural control‐specific component, including dynamic single‐leg postural control exercises, such as single‐leg stances while moving a ball, single‐legged ball throws and catches, and single‐legged light wrestling activities [29, 30, 31]. Based on the results of this study, identifying and fixing limb asymmetries in dynamic postural control might prove beneficial in terms of preventing noncontact ACL injuries.

It is important to acknowledge that dynamic postural control tests such as the mSEBT challenge athletes not only regarding proprioception but also strength and range of motion [32, 33]. Thus, training programs aimed at preventing ACL injuries should also include exercises correcting potential between‐limb differences in strength and range of motion such as single‐leg squat variants. Between‐limb asymmetries in strength and range of motion have previously been associated with increased lower extremity injury risk in certain studies [34, 35, 36].

In addition to the association between limb asymmetry on the mSEBT and the risk of injury, the hazard ratios on the single‐leg drop‐down test could possibly point toward an increased risk for injury with higher asymmetry results for average mediolateral and anteroposterior velocities, as well as square side lengths, but these relationships were not statistically significant. This may be due to the relatively small number of ACL injuries and the resulting lack of statistical power. Although no conclusions can be drawn based on the single‐leg drop‐down test results, these nearly significant findings further support the idea that asymmetry might be a risk factor for ACL injuries in female youth athletes.

No relationships were found between any of the variables analyzed based on the single‐leg stance test, which is considered a measurement of static postural control and injury risk. This lack of a relationship with injury risk may result from the test being unable to challenge the postural control of the youth athletes adequately. Performing the single‐leg stance test eyes‐closed could have provided the necessary challenge to better identify problems in static postural control. Previous publications by Wikstrom and others on dynamic postural control made an argument that there seems to be no association between dynamic and static postural control test results [6]. As noncontact ACL injuries occur during activities that challenge dynamic postural control, postural control tests measuring static postural control may be inadequate in terms of identifying the risk of injury.

This study did not find associations between mSEBT bilateral limb mean results and risk of injury. Since the inception of this study protocol, recommended mSEBT reach directions have changed to include anterior and posterolateral directions in addition to the posteromedial direction that was included in this study [37]. It is possible that use of the current recommended reach directions and test procedures could have yielded more associations between mSEBT and risk of noncontact ACL injury, especially considering the earlier findings on anterior asymmetry and lower extremity injury risk [23, 37].

### 4.1 Strengths and Limitations

This study was the first to investigate the relationship between dynamic, static, and stabilization measurements of postural control and noncontact ACL injury risk in female youth team sports. This investigation included the prospective registration of ACL injuries and individually collected exposure data, which allowed the use of Cox regression in the analysis. The follow‐up period extending up to 3 years is another strength of the study.

Some limitations should be acknowledged. Although the reliability of the SEBT and static measurements of postural control, such as measuring postural swaying using tests like the single‐leg stance balance test, has been previously established, no reliability studies were found for the single‐leg drop‐down test [15, 23, 24]. However, the reliability of a similar test using time to stabilization, as measured by the force platform, ranged from moderate to excellent [38]. As the population considered in this study consisted of only female youth basketball and floorball athletes, the generalizability of these results is limited. Thus, the results might not be applicable to men, older populations, or athletes competing in other types of sports. As the number of noncontact ACL injuries was relatively low (12 cases), our study may not have had sufficient power to identify weaker associations [19].

As previous ACL injury has been associated with impairments in postural control, regression analyses were also redone by excluding the three athletes with previous ACL injury, but this did not affect the results, and the original approach was retained [39].

### 4.2 Perspective

Increased between‐limb asymmetry in the posteromedial direction and composite score of the mSEBT were identified as risk factors for noncontact ACL injury in youth female basketball and floorball athletes. Identifying and correcting potential asymmetries in dynamic postural control by training consisting of unilateral proprioception, strength, and range of motion exercises might prove beneficial in decreasing the risk of noncontact ACL injury.

## Ethics Statement

This study was conducted in accordance with the Declaration of Helsinki and approved by the Ethics Committee of the Pirkanmaa Hospital District, Tampere, Finland (ETL‐code R10169).

## Consent

The athletes signed a written informed consent form, with a parent's or guardian's consent being acquired from athletes under the age of 18 before taking part in the study.

## Conflicts of Interest

The authors declare no conflicts of interest.

## Data Availability

The data that support the findings of this study are available on request from the corresponding author. The data are not publicly available due to privacy or ethical restrictions.
